# Oral Sepsis and Children’s Diseases

**Published:** 1915-06

**Authors:** 


### ORAL SEPSIS AND CHILDREN'S DISEASES.

The relationship of oral sepsis to many diseases has been well substantiated. The most eminent and scientific authorities in both the medical and dental professions have agreed that a definite connection exists. There is no group of diseases, however, in which the connection is more marked than in the group known as children's diseases.

There are certain well-defined facts in connection with the common infectious diseases of childhood. The focus of infection in all cases is in or near the mouth. It is recognized that the infection is carried in the secretions of the mouth, nose and throat. The prevalence of these diseases is very much reduced when the schools close for the summer vacation, and increases when they open again in the fall. These diseases occur as a rule in childhood during the period in life when, owing to neglect, the mouth is in the worst possible condition.

It is also a significant fact that during childhood there are conditions obtaining in the mouth that are peculiar to that period. The physiological resorption of the roots of the deciduous teeth is responsible for the fact that at all times during the shedding process there are open apices of roots in the mouth of the child. When caries reaches the pulp chamber, in such cases, there are direct openings from the mouth to the tissues at the apices of the roots, open avenues of infection. The same condition is found in the first permanent molars, where decay has reached the pulp chamber before the completion of the roots.

We have, then, in the mouths of the majority of children a combination of conditions which does not obtain at any other period in life—a neglected, filthy and diseased condition of the teeth and mouth, accompanied by direct avenues of infection leading to the deeper tissues.

It is a reasonable deduction that the prevalencee and spread of the infectious diseases of childhood are due directly to the general condition of children's mouths, and this view is held by a number of eminent health authorities.

It has remained, however, for Dr. Keys, a member of the dental profession of Boston, to collect statistics which prove the point conclusively. In St. Vincent's Orphan Asylum in that city the average number of cases of infectious disease, per year, was about 103. A dental clinic was established in November, 1910. The results bear out what in theory seems reasonable, infectious disease has been eliminated from the Asylum. There has been no more valuable contribution to the subject of preventive medicine than the publication of the following record by Dr. Keyes:

| | 1907-1908 | 1908-1909 | 1909 Nov. 1910 | Nov. 1910 Apr. 1911 | Apr. 1911 May 1912 | May 1912 May 1913 | May 1913 May 1914 |
|---|---|---|---|---|---|---|---|
| Diphtheria | 6 | 2 | 1 | 0 | 0 | 0 | 1 |
| Mumps | 8 | 3 | 10 | 4 | 0 | 0 | 0 |
| Scarlet Fever | 17 | 8 | 12 | 8 | 0 | 0 | 0 |
| Pneumonia | 3 | 5 | 4 | 6 | 0 | 0 | 0 |
| Measles | 24 | 50 | 40 | 25 | 0 | 0 | 0 |
| Tonsilitis | 19 | 16 | 8 | 3 | 0 | 0 | 0 |
| Whooping Cough | 7 | 2 | 2 | 0 | 0 | 0 | 0 |
| Chicken Pox | 15 | 17 | 10 | 6 | 0 | 0 | 0 |
| Typhoid | 0 | 0 | 0 | 0 | 0 | 0 | 0 |
| Croup | 4 | 0 | 0 | 0 | 0 | 0 | 0 |
| Spinal Meningitis | 0 | 0 | 0 | 0 | 0 | 0 | 0 |
| Scarlatina | 0 | 0 | 0 | 0 | 0 | 0 | 0 |
| Bright's (Acute) | 0 | 0 | 0 | 0 | 0 | 0 | 0 |
| Hemorrhage | 0 | 0 | 0 | 0 | 0 | 0 | 0 |
| Tuberculosis of Eye | 0 | 0 | 0 | 0 | 1 | 0 | 0 |
| Tuberculosis of Lungs | 0 | 0 | 0 | 0 | 1 | 0 | 0 |
| | 103 | 103 | 87 | 52 | 2 | 0 | 1 |

The single case of diptheria recorded in the last column occurred in a new girl. Examination of her mouth showed great need of dental treatment. One of the six cases of measles was contracted before or immediately after admittance to the Asylum and spread to five other children.—*Oral Health.*

